# Synergising Public Health Concepts with the Sendai Framework for Disaster Risk Reduction: A Conceptual Glossary

**DOI:** 10.3390/ijerph13121241

**Published:** 2016-12-14

**Authors:** Suzanne Phibbs, Christine Kenney, Christina Severinsen, Jon Mitchell, Roger Hughes

**Affiliations:** 1 School of Public Health, Massey University, Palmerston North Campus, Palmerston North 4442, New Zealand; c.a.severinsen@massey.ac.nz; 2 Joint Centre for Disaster Research, Massey University, Wellington 6140, New Zealand; c.kenney@massey.ac.nz (C.K.); J.Mitchell1@massey.ac.nz (J.M.); 3 School of Public Health, Massey University, Wellington Campus, Wellington 6140, New Zealand; R.Hughes@massey.ac.nz

**Keywords:** public health, disaster, Sendai Framework, inequity, community development, inverse care law, prevention paradox

## Abstract

The Sendai Framework for Disaster Risk Reduction (2015) is a global strategy for addressing disaster risk and resilience that has been ratified by member countries of the United Nations. Its guiding principles emphasise building resilience through inter-sectoral collaboration, as well as partnerships that facilitate community empowerment and address underlying risk factors. Both public health and the emergency management sector face similar challenges related to developing and implementing strategies that involve structural change, facilitating community resilience and addressing individual risk factors. Familiarity with public health principles enables an understanding of the holistic approach to risk reduction that is outlined within the Sendai Framework. We present seven concepts that resonate with contemporary public health practice, namely: the social determinants of health; inequality and inequity; the inverse care law; community-based and community development approaches; hard to reach communities and services; the prevention paradox; and the inverse prevention law. These ideas from public health provide a useful conceptual base for the ”new” agenda in disaster risk management that underpins the 2015 Sendai Framework. The relevance of these ideas to disaster risk management and research is illustrated through drawing on the Sendai Framework, disaster literature and exemplars from the 2010–2011 earthquakes in Canterbury, New Zealand.

## 1. Introduction

In 2015 three landmark UN agreements were enacted with: (1) The Sendai Framework for Disaster Risk Reduction 2015–2030 (Sendai Framework) adopted in March 2015 in Sendai, Japan by 187 United Nations (UN) member states; (2) The Sustainable Development Goals (SDGs) agreed in September 2015 in New York, USA by 193 countries; and (3) The Paris Agreement on Climate Change, signed by 195 countries in December 2015 at the Paris Climate Conference (CoP21). The first, The Sendai Framework for Disaster Risk Reduction 2015–2030, aims to reduce disaster losses in lives, livelihoods, and health by a series of agreed actions and builds on the previous Hyogo Framework for Action 2005–2015 [1]. The goals and actions of the Sendai Framework for Disaster Risk Reduction [2] specifically include:

“The substantial reduction of disaster risk and losses in lives, livelihoods and health and in the economic, physical, social, cultural and environmental assets of persons, businesses, communities and countries… through the implementation of integrated and inclusive economic, structural, legal, social, health, cultural, educational, environmental, technological, political and institutional measures that prevent and reduce hazard exposure and vulnerability to disaster, increase preparedness for response and recovery and thus strengthen resilience”.

(p. 7)

The four priority areas for action include: “understanding disaster risk; strengthening disaster risk governance to manage disaster risk; investing in disaster risk reduction resilience; and enhancing disaster effective response to ‘Build Back Better’ in recovery, rehabilitation and reconstruction” (p. 9) [2]. All three landmark UN agreements, as well as the goals and priority areas for action within the Sendai Framework specifically focus on Disaster Risk Reduction (DRR) outcomes through a set of Disaster Risk Management (DRM) actions. 

The “new” approach to Disaster Risk Management (DRM) emerged in the 1990s and has developed alongside the Hyogo [3] and Sendai [2] Frameworks for Disaster Risk Reduction. The “new” DRM is characterised by a move away from a wholly response and recovery focus to an approach that includes reduction, readiness, response and recovery [4]. Features of the “new” approach include a shift from a single focus on top-down prevention initiatives and centralised response to one that also includes reducing existing risks and building resilience to current and future risks. Key characteristics of the “new” emergency management include: a focus on being proactive as well as reactive; balancing the need for command and control in an acute emergency situation with a partnership approach to disaster preparedness, planning and risk reduction; building community capacity prior to a disaster alongside identifying and responding to need post-impact; and combining individual disaster preparedness with legislative change and bureaucratic intervention in order to facilitate hazard mitigation and reduce underlying disaster risk factors. Familiarity with public health principles enables a synergistic understanding of the holistic approach to risk reduction that is outlined within the “new” emergency management and in the Sendai Framework. 

In this article seven key concepts within public health are discussed. The application of these ideas within the disaster risk and emergency management fields is illustrated using examples from the Sendai Framework for Disaster Risk Reduction [2], the disaster literature as well as the 2010–2011 earthquakes in Canterbury, New Zealand. Both contemporary public health and the field of Disaster Risk Management take into account the underlying social, economic, political and environmental determinants of health [2,3,5]. Consideration of this broad set of influences and determinants, many of which sit outside the disaster response context, ensures that key conditions and factors which determine and influence population-level outcomes following disaster are taken into account. Public health is directed at reducing health inequality and inequity between population groups [6]. The distribution of determinants and outcomes across populations is also a similar consideration in disaster epidemiology [7]. Post-disaster outcomes can be improved by using an equity approach that focuses on reducing social and economic vulnerabilities prior to a disaster. The inverse care law [8], which states that populations that require the most care often receive the least care and to a lesser standard, also has implications for disaster response and recovery planning and resourcing. The inverse care law may be ameliorated through attention to building community capacity in the areas of disaster preparedness, planning and response. 

Within the public health literature there is a distinction between top down community-based approaches and bottom up community development approaches [9]. These different ways of working with communities have implications for how disaster and emergency management organisations develop relationships with communities to prepare for emergencies. Within public health the term “hard-to-reach populations” typically refers to marginalised and/or vulnerable groups that fall outside of the mainstream service provision [10]. In contrast, the concept of hard-to-reach organisations turns the lens from seeing the characteristics of particular groups as the problem to exploring how organisational policies and practices may work to marginalise and exclude [11]. In this view, population health is a collective responsibility requiring community partnerships as well as collaboration and accountability across all levels and sectors. The “new” DRM also includes an approach to disaster preparedness, planning and response that is multi-level, and incorporates inter-sectoral action in a variety of settings including those that are traditionally outside of the formal emergency management sector [2]. Public health works at the population or population group level. This means action focuses on the whole population, rather than at-risk individuals and groups. We argue that strategies that target the entire population have the greatest potential to reduce disaster risk across all socio-economic groups. Lastly, the prevention paradox [12] has identified that having a sole focus on targeting individuals and groups at high risk will have limited effect at a population level and may even increase inequalities. Intervention generated inequalities are likely to be magnified by mass media campaigns and strategies that require voluntary action and/or co-funding.

This paper arose out of discussions of synergies between public health concepts and the holistic approach to disaster risk reduction that is outlined in the Sendai Framework [2]. The intention of the paper is to share scientific knowledge from public health in order to open up a conversation about the conceptual base that underpins the Sendai Framework for Disaster Risk Reduction. It is hoped that aspects of this glossary will be useful in training future disaster researchers and emergency management practitioners about the broad multi-level and multi-sectoral techniques for disaster risk reduction that is the basis for contemporary public health and the “new” DRM approach. 

## 2. Materials and Methods

A literature search was conducted for English language, peer reviewed journal articles using the academic database ‘*Discover*’ as well as *Google Scholar*. Key words were used that related to the public health concepts discussed in this article. The following search terms were entered: “prevention paradox” and “disaster risk reduction”. From this search one journal article was identified within the disaster literature that linked the prevention paradox to resilience [13]. The search terms “inequity” and “post disaster” returned several articles related to gender, ethnicity and the distribution of resources in the wake of disaster [14,15,16,17]. The search terms “inverse care law” and “disaster response” identified literature that focused upon emergency care post-impact [18], as well as access to primary health care [19] and housing [20] in recovery. Additional articles, that focus upon inequalities in the distribution of resources post-disaster [21,22,23], that could be used to illustrate arguments related to the inverse response law, were identified using the search terms “inequalit*” or “unequal” “resource distribution” and “disaster”. A search for the terms “inverse prevention law” and “disaster preparedness” did not return any results. We were unable to find any literature that included two or more concepts from public health in the same article. The remaining public health and disaster literature used to support arguments presented in this paper is taken from classic texts within public health [8,24] as well as knowledge gained from teaching and publishing in the public health and disaster areas. A limitation of this paper is that a full discussion of the controversies, and potential knowledge gaps, associated with each of the seven public health concepts has not been provided, however, these debates have been covered elsewhere [6]. 

Two of the contributing authors to this paper were principal investigators for two separate qualitative research projects conducted in Christchurch following the Canterbury earthquake sequence. We use case studies from these research projects and examples from the disaster literature to illustrate public health concepts discussed in this article. Both research projects were approved by the Massey University Human Ethics Committee (MUHEC). The Māori resilience research project MUHEC approval number is Southern B 12/31 and the disability research approval number is Southern A 12/18.

The first of these projects [25] was a three year study conducted with Māori tribal and community stakeholders who facilitated the recruitment of research participants. Semi-structured, face-to-face and group interviews were conducted in 2012 and 2013 with 70 Te Rūnanga o Ngāi Tahu employees, as well as Ngāi Tahu kaumātua (elders), rūnanga (sub-tribe) members and Māori community volunteers who were involved in the Ngāi Tahu-led response to the Canterbury earthquakes. Overarching themes included: specific tribal and Māori organisational recovery initiatives; ways in which cultural beliefs, values and practices facilitated disaster risk reduction and mitigation; recommendations for the formal emergency management sector as well as disaster preparedness planning within Māori organisations and communities. Data analysis drew on abductive research strategies [26] which enabled participants to be involved in developing the analytical descriptions and explanations of cultural factors that facilitated disaster risk reduction and management. All participants signed a consent form. Audio-taped interviews were transcribed verbatim and analysed in paragraph format to ensure that data interpretation was accurate. Key themes were identified using thematic analysis [27] the researchers also liaised with Māori participants to ensure that findings reflected the participants’ experiences and that any discrepancies in understandings were addressed. Community engagement enabled the capture of Māori understandings and practices associated with risk reduction and mitigation, disaster preparedness, response and recovery. In order to ensure participant confidentiality anonymised data was used in subsequent publications from the research. 

The second research project [28,29] was a one-year project conducted in 2012 with 23 disabled people living in Christchurch during the earthquakes and four agency representatives who talked about how the earthquakes had impacted upon their organisation and clients. Overarching themes included individual, community and organisational preparedness and response. The efficacy of welfare centres for disabled people as well as health issues, housing, mobility and information needs [30]. Audio-taped interviews lasting up to 90 min took place in participants’ own homes. The same interviewer conducted all of the interviews, reviewed the information sheet, ensured informed consent through explaining to participants their rights and answering any questions about the research. All participants signed a consent form. Audio-taped interviews were transcribed verbatim and participants were given pseudonyms to ensure confidentiality. The qualitative interview material was analysed using thematic analysis [27], whereby interview transcripts were manually coded and arranged into themes. Themes were then analysed in relation to relevant literature within the areas of disability and disaster response and recovery. 

Interview transcripts from both projects were reviewed and narratives that highlight synergies between the named public health concepts and the principles outlined in the Sendai Framework for Disaster Risk Reduction [2] were identified. Further to consensus amongst the authors, narrative extracts from participants’ talk that illustrate the relevance of public health concepts to the Sendai Framework and Disaster Risk Reduction were selected and applied as exemplars to reinforce arguments presented. Fuller discussions of the research methodologies and overviews of the research findings have been published elsewhere [25,28,29,31]. The article should not be considered to be a definitive analysis of public and private sector responses to the Canterbury earthquake sequence. This paper should be read as a conceptual glossary that uses examples from the Christchurch research to illustrate themes at work within different sections. 

## 3. Results

### 3.1. Synergising Public Health Concepts with the Sendai Framework for Disaster Risk Reduction

Christchurch is located in the Canterbury region of New Zealand’s South Island. It is the country’s second largest city with an estimated population of 340,000 [32]. On 4 September 2010 at 4:35 a.m., a shallow, magnitude 7.1 earthquake occurred in Canterbury, heralding a cycle of earthquakes that caused widespread damage within the region. On the 22 of February 2011 a magnitude 6.3 earthquake centred under Christchurch devastated the city, killing 185 people and injuring a further 7171 [33]. Christchurch is a coastal city that is mainly flat with its southern suburbs located on the Port Hills. The central city, hillside and coastal Eastern suburbs received the most damage with the latter being affected by severe liquefaction. The 22 February earthquake resulted in damage to nearly three quarters of the housing within the region, prolonged loss of power, water and sewerage, damage to roads and severe destruction in the central business district which was wholly or partially cordoned off until the 30 June 2013 [33]. Christchurch residents endured more than 12,000 aftershocks throughout 2010–2011, four of which were magnitude 6.0 or greater. Drawing upon examples from Christchurch, ideas from public health are applied to how people involved in the emergency management sector develop relationships with communities as well as understand risk and vulnerability to disaster. 

According to the World Health Organisation public health refers to “all organised measures (whether public or private) to prevent disease, promote health, and prolong life among the population as a whole. Its activities aim to provide conditions in which people can be healthy and focus on entire populations, not on individual patients or diseases. Thus, public health is concerned with the total system and not only the eradication of a particular disease” [34]. 

Public health is a field that includes multiple domains and disciplines. It is concerned with the population as a whole and combines diverse strategies from across the micro (individual), meso (community) and macro (structural) levels of social systems in order to improve health outcomes. Structural approaches, which identify the macro level determinants that contribute to poor outcomes, have the greatest potential to reduce risk and improve overall population health. Disaster risk is also socially patterned and disproportionately experienced in disadvantaged communities. Improving outcomes post disaster involves directing action upstream to influence the political, social, environmental and economic determinants of disaster vulnerability. 

### 3.2. Social Determinants of Health

Public health takes into account the underlying social, economic, political and environmental determinants of health [5,35]. These determinants link to priority one in the Sendai Framework which is related to understanding disaster risk [2]. The social determinants of health include, but are not limited to, income, employment, occupation, education, housing, area of residence, social exclusion, transport, food and access to health care [5,35,36]. These factors are also present in the emergency management and disaster literature as determinants of vulnerability [2,3,5,37,38]. Consideration of this broad set of determinants, many of which sit outside the traditional remit of the emergency response sector, ensures that key conditions and factors which determine and influence population level outcomes following disaster are taken into account [5]. According to this perspective vulnerabilities prior to a disaster, such as surviving on a low income [39], exposure to food insecurity, or living in crowded housing, will be exacerbated after a disaster. The Hyogo Framework for Action [3] set the agenda for emergency preparedness, planning and response until the year 2015. Recent reviews of advances made since the ratification of the Hyogo Framework in 2005 identified that the least progress had been made in the action area relating to reducing underlying risk factors in which social vulnerability is one of the key areas of concern [2]. Low socio-economic status and living in low income neighbourhoods are risk factors for earthquake vulnerability and the erosion of resilience during the disaster recovery phase [37,40,41,42]. Financial hardship increases stress, erodes resilience and prolongs dependency [42]. Following the February 2011 Canterbury earthquake the link between socio-economic status and increased vulnerability was highlighted in the areas of Christchurch that were impacted and in the narratives of participants in the Māori research referenced above. 

Although the Canterbury earthquake sequence had a catastrophic impact on the people and wider environment, the Eastern suburbs of Christchurch, including the lower socio-economic areas of Aranui, Bexley, Wainoni [43,44] and Dallington, were among the most severely affected areas [45] that had the poorest outcomes [37]. To underscore the degree of hardship that existed in these areas of the city prior to the earthquakes; Aranui had a decile rating of 10 on the 2006 New Zealand Deprivation Index [46] meaning the suburb is among the 10% of New Zealand areas that are the most deprived. In 2006 the decile rating of Bexley was 9, Wainoni 8 and Dallington 6 [44,46]. Commenting on the situation in general for families living in Aranui, and on the situation of one family in particular, a participant in the Māori research who worked within the community following the February earthquake stated:

…in the Eastern suburbs over in Aranui, just...no power, no water, no toilets, no food in the cupboards basically, no money because they couldn’t [get money]—the “EFTPOS” machine wasn’t working, none of the shops were open. [The father of one family] had no petrol in his car… so they were all at home. But there they are huddled around... they’d got a fire going in some tires outside the front door, and the kids were sleeping in the lounge and on mattresses on the floor, just terrified and nothing to eat… we went back the next day and nothing was better for them….

(KR, 2012)

All of the communities in Christchurch were impacted by the earthquakes with most suburbs experiencing similar disruptions to infrastructure such as power, water, sewerage, roads, shops and Electronic Funds Transfer at Point Of Sale [EFTPOS] facilities. However, in many lower socio-economic areas a lack of household resources, that could be used to sustain a family immediately after a major seismic event, underscores the importance of increasing resilience through addressing the social and economic determinants of vulnerability. Households within deprived neighbourhoods face a double burden following disaster. When a similar lack of basic resources is repeated within households in the same geographical area, community resilience is eroded and dependency is increased [42]. In public health the combined impact of negative community characteristics on individual outcomes is called the *neighbourhood effect*. According to the neighbourhood effect literature [47] poor people who live in disadvantaged neighbourhoods experience worse outcomes across a range of individual measures, such as educational levels, substance abuse and exposure to violence, than poor people who live in wealthier neighbourhoods. The neighbourhood effect implies that post disaster vulnerabilities, such as lack of access to basic necessities including food and water as well as the money and transport to acquire them, will be magnified for deprived communities.

The social determinants of health, such as income, education, occupation and housing are often located at the individual level, however social determinants are also influenced by the social structure of society [48,49]. These determinants of health refer to the way that resources are distributed within society. Structural determinants of health include wealth and income inequality, housing availability and affordability as well as public policy in areas such as taxation, land use planning and regulations governing workplace safety. 

### 3.3. Inequality and Inequity

Within public health a clear distinction is made between *health inequality* and *health inequity* [6]. Health inequality is a descriptive term that refers to measurable differences in health outcomes between population groups. Health inequity is a normative term that refers to those measureable differences in health outcomes that arise out of injustice and are therefore potentially avoidable, unnecessary or unfair [6]. Measuring and reporting upon the economic, social, cultural, educational, health and heritage impacts following a specific disaster event are related to the first priority area with the Sendai Framework for Disaster Risk Reduction [2]. The public health distinction between inequality and inequity may also be applied to documenting outcomes within the disaster field. The Canterbury earthquake sequence caused considerable damage to the built environment within the region. Unequal access to resources, such as housing, post disaster is not inequitable as people do not have control over outcomes that result from a natural hazard event [50]. Damage to housing, combined with disruption to utilities such as water, electricity and sewerage, meant that there was a high demand for emergency accommodation at Civil Defence welfare centres [51]. Emergency shelters that do not meet the needs of disabled people are an example of inequitable access to accommodation in the aftermath of a disaster. The following narrative, from a participant in the disability research, who had cerebral palsy and used a power-chair, illustrates how inequitable access to emergency accommodation for disabled people following the February earthquake [30,52] has very real consequences for individuals:

I was in my bedroom and my bedroom wall collapsed… I couldn’t get out my door… so my flatmate had a carer in there and she had to create a pathway for me and I knew as soon as my bedroom wall collapsed I wouldn’t be living at that house. …I couldn’t get down there [the local Civil Defence welfare centre] by myself so I had to get one of my neighbours to push me down there because of all the liquefaction and the roads were bent... But I had to stay with my neighbour that night on a two seater couch because the [welfare centre] said … because my neighbour had an impairment as well, they said that they couldn’t accommodate people with disabilities… they didn’t even ask us about “oh what supports do you guys need”, we just got told, they said “we can’t accommodate you”. [Which was] insulting, because they just saw the impairment and not the person… Yep, during the earthquake it happened quite a bit especially during like the first week after the February earthquakes….

(Rangimarie, interviewed in 2012)

In this localized example, access to shelter was inequitable as preferential access to the welfare centre was extended to people who were able bodied. Inequity results from structural factors that are beyond the control of individuals. Structural inequities in access to material resources, such as housing, are not discrete but tend to persist over time [50] suggesting that these inequities are present prior to a disaster. A structural approach to disaster risk reduction employs legislative change, as well as adjustments to national and regional government policy settings, in order to address underlying risk factors which create the conditions of social vulnerability post disaster. The way in which structural disparities in access to resources pre-disaster can create problems for vulnerable populations in the aftermath of a disaster may be illustrated in relation to disability accessible housing. 

Prior to the Canterbury earthquakes, much of the rental housing stock in Christchurch was older, poorly maintained and did not comply with contemporary earthquake-resilient building codes [53]. Following the Canterbury earthquakes housing availability was reduced and the number of affordable rental houses decreased markedly. A lack of rental accommodation was associated with increased rents and higher rates of unmet housing need [33,53]. Housing availability was further restricted for people who needed accessible housing as there are fewer accessible houses within the general housing stock [20]. In addition, a significant percentage of the disability accessible social housing within the city was provided by the Christchurch City Council and the State provider Housing New Zealand. The majority of this housing was constructed in the 1970s and 80s and was not earthquake-resilient [53]. In the aftermath of a disaster housing inequality may be measured in relation to the total number of people who were in severe housing need. However, this need becomes structurally inequitable when lack of access to housing is a result of an unequal distribution of accessible housing within the population [20]. Ensuring that social housing is upgraded to comply with contemporary building standards, that ongoing maintenance is provided and that the housing needs of the disabled are met are examples of how disaster preparedness planning includes actions by organisations that are traditionally outside of the domain of the disaster response and emergency management sector. 

The public health discipline aims to reduce structural inequality and inequity between population groups. The distribution of outcomes across populations is also a key consideration in the disaster field. Pre-disaster it is possible for the public and private sector to work to improve population outcomes post-disaster through focusing on achieving equity. Te Rūnanga o Ngāi Tahu is the organisation that oversees the commercial interests of the Ngāi Tahu Iwi. In 2016 Ngāi Tahu had an asset base of over 1.2 billion NZ dollars [54]. Ngāi Tahu, as the resident Māori tribe in Christchurch, is leading community development initiatives post disaster that focus upon addressing cultural exclusion, inadequate accommodation and high unemployment rates among Māori. These initiatives are exemplars of using an equity approach to facilitate social resilience through strengthening community, and reducing social and economic vulnerability to subsequent disasters. Various equitable accommodation programmes have been established, including a partnership between the Canterbury Community Trust and Ngāi Tahu, that is developing social housing [55]. Some tribally owned rural land holdings, although economically viable as farms, have also been designated for urban development to help address the acute shortage of suitable housing land. Equally, purchase prices for Māori land incorporated into housing developments at the time of the 2010 earthquakes remained fixed at pre-September 2010 rateable values despite a rapid increase in the cost of both housing and residential land at the time. According to the Ngāi Tahu leader the rationale for freezing tribal land prices in 2010 was that profiteering from the misfortune of others is incompatible with tribal cultural values and that all Cantabrians should have equitable access to owning a home [56]. 

Māori workforce development is being fostered through He Toki ki te Rika (the Māori Pre-Trade Training Scheme) and He Toki ki te Mahi (Māori Trade Apprenticeships Programme) initiatives led by Te Rūnanga o Ngāi Tahu in partnership with the Christchurch Polytechnic and the Hawkins Construction Group [57]. This model leverages the existing strengths, knowledge, experience and capabilities of partner organisations to up-skill Māori for the Canterbury rebuild. The initiatives offer youth residing in socio-economically deprived areas a foundation for entry into employment through apprenticeship pathways into the construction industry. The consistently positive outcomes from the schemes are remarkable considering that Māori unemployment rates tend to be twice the national average (12.3% compared to 4.4% for Europeans in 2014 [58]). Māori youth aged 15–24 also experience the highest levels of labour market, training and educational disengagement [59] (in 2016, 21.1% compared to 9.2% for Europeans [60]). Five years on from the earthquake sequence, these successful programmes have contributed to the development of an additional public private partnership that fosters agricultural workforce development. Whenua Kura, is an initiative developed by Ngāi Tahu Property, Te Tapuae o Rehua and Lincoln University. The programme engages Māori in studying toward university qualifications specialising in land-based studies, work placements on Ngāi Tahu farms, a Māori approach to learning, as well as guidance and support through to employment. The initial student intake commenced in 2014, with subsequent intakes in 2015 and 2016. To date course completions remain high [57,61]. Using a cross-sector approach that ensures the use of traditional indigenous knowledge in the development of plans, strategies and policies to reduce disaster risk is also recognised in the first priority area of the Sendai Framework [2]. 

The examples of building community resilience through proactively addressing factors that create vulnerabilities and contribute to poor outcomes post-disaster, such as unemployment, poverty and poor housing, presented in this section illustrate how disaster risk reduction operates at multiple levels, incorporating inter-sectoral action in a variety of settings that are not traditionally included within the area of disaster preparedness planning. 

### 3.4. The Inverse Care Law

The *inverse care law* refers to the idea that “(t)he availability of good medical care tends to vary inversely with the need for it in the population served” (p. 405) [8]. According to the inverse care law those who require the most care actually receive the least and to a lesser standard. Improving response efficacy and reaching vulnerable populations is a focus of priority four of the Sendai Framework [2] which emphasises enhancing disaster preparedness for effective response. Drawing upon the principles within the inverse care law, we propose an inverse response law that relates to access to emergency services and supplies in the aftermath of a disaster. The inverse response law suggests that those people in lower socio-economic groups, who tend to be disproportionately impacted in a disaster, tend to receive the least help and to a lesser standard [21,22,23]. The inverse response law, whereby disadvantaged and/or marginalised groups may have a greater need but receive less attention in the formal disaster response was implicitly recognised by participants in the Māori research that talked about the Māori response to the February earthquakes:

I asked the Māori community if we could include the Asian and migrant community, because they would be outside [of the mainstream response], to which I got an immediate agreement.

(SMS, 2012)

And

Our goal was really about helping people help themselves by actually seeing that they were able to get the things they were entitled to, and that was the first priority… the people that were falling out of the safety net if you like, that the government had put in place.

(SM, 2012)

And

…the focus for us [the Māori response] was people that often end up marginalised … [that] mainstream doesn't necessarily cater for…”.

(OD, 2012)

Related to the inverse care law is the idea that there are large social inequalities in the patterning of vulnerability and distribution of resources post disaster [17,21]. Higher income groups know how to make better use of services, they are more critical, tend to receive more attention and are able to get better access to resources and equipment than low income groups [8,21,62]. An example of inequalities in the distribution of resources in post-disaster Christchurch could be seen in the distribution of portable toilets between the high income suburb of Westmorland and the Eastern suburbs as discussed by a participant in the Māori research:

I think it was the Friday after the [February] earthquake, I [had] a meeting and right at the bottom of Westmorland hills there were about 20 Port-a-loos all lined up. So we go up the hill to [names place] and we say, ‘got a bit of a walk to the Port-a-loo at the bottom of the hill mate’, and he says ‘ah no full sewerage here... nearly everyone on the hill’s rung them—why did you put them there for, they’re not needed, we’ve got sewerage’. They stayed there for weeks, while the people in the Eastern suburbs had nothing. Poor planning, poor follow up!

(SMS, 2012)

Another participant from the Māori research also noted the absence of portable toilets in the Eastern suburbs:

The whole of [names street], which stretches between two bridges and takes up about 2 km of roadway, a lot of houses, not one toilet was delivered. We went without; there was not one Port-a-loo in sight for the whole period [six weeks]. Yep, there was nothing on our street at all, and the surrounding streets, so it made life hugely difficult.

(HD)

These Māori participants, who were known to each other, noted the oversupply of portable toilets in a wealthier suburb that was marginally affected and compared this with a lack of portable toilets in the low income areas that were severely impacted. Observations regarding the portable toilets provided tangible evidence that confirmed the participants’ impressions that post-impact the lower socio-economic areas were being poorly served by the formal emergency response. 

Post-impact portable toilets were deemed assets of strategic importance, commandeered and distributed through the formal emergency response sector [63]. A lack of portable toilets in the low socio-economic area of Aranui was noted in a media report post-impact [64] and in research that mapped the distribution of portable toilets in Christchurch in March of 2011 which identified inequitable access to portable toilets in the Eastern suburbs [63]. The inverse care law also states that disadvantaged groups are least likely to engage with health care services and to receive formal training [8]. In the emergency management sector, the inverse care law may be minimised by ensuring that ‘lay’ emergency response training reaches those disadvantaged or marginalised groups who are at greatest risk and will need high levels of support post disaster. Working with a diverse range of communities is one way of ensuring that disaster preparedness initiatives will reach groups that are typically outside of the mainstream response. 

### 3.5. Community-Based and Community Development Approaches

Public health practice requires a range of sectors and groups to work collaboratively, and promotes partnerships with community [65]. Attention to multi-sectoral cooperation, and the development of partnerships with key stakeholders and communities, is a consideration within priority area two in the Sendai Framework for Disaster Risk Reduction which focuses upon strengthening disaster risk governance [2]. The public health literature distinguishes between top down community-based and collaborative bottom-up community development approaches [9,66]. The “new” Disaster Risk Management is also involved in building community resilience [4]. These different ways of working with communities have implications for how organisations involved in disaster risk reduction and emergency preparedness planning develop relationships with and work to prepare communities for disaster. In community-based health promotion, problems, targets and actions are defined by the sponsoring body. The notion of community is relatively unproblematic, with community settings being viewed as venues for interventions that largely target the individual. In these top-down community-based interventions, activities are mainly health, or in this case disaster preparedness, oriented [66]. Community-based initiatives tend to be single issue focused and time-limited, discontinuing once the sponsoring body has withdrawn (for an example of community-based disaster preparedness planning and response see [4]). 

Prior to the emergence of the “new” Disaster Risk Management approach disaster preparedness and response initiatives followed a similar top-down, command and control model in which trained experts provided advice to community members who are regarded as passive recipients of information. More recently, the “new” Disaster Risk Management has moved from a community-based approach to also include a community development perspective which seeks to empower communities and play an active role in their advancement. The formal emergency management sector has also started to acknowledge the value of voluntary community focused responders in disaster management, preparedness and response [67]. Community development is included in priority area one within the Sendai Framework for Disaster Risk Reduction [2] which focuses upon enhancing collaboration with people at the local level. 

In a community development perspective, problems, targets and actions are defined by the community which is recognised as a complex entity that is changeable and subject to power imbalances. Actions are aimed at supporting the community through focusing upon capacity building and empowerment. The target of the intervention may be the community itself or structures, services or policies that impact negatively upon the community by creating vulnerabilities [66]. Activities may be broad-based, targeting wider factors which are associated with negative social outcomes, such as discrimination [68,69], poverty or crime [70], thereby providing indirect disaster resilience outcomes such as facilitating community empowerment and enhancing social capital. One example of a community development approach can be seen in the disaster preparedness initiatives untaken by the Wellington based marae [Māori Community Centre] Ngā Hau e Whā o Paparārangi following their experience of receiving evacuees from the February 2011 Canterbury earthquake:

After the Christchurch earthquake we sat down with a number of other marae [Māori community centres], we were sponsored by the Hutt [City] Council, and we ended up with 10 marae dedicated to improving their standards. Not only have we tested our [emergency] procedures… we have expanded to include the doctors, the pharmacy, the local community centre, the ham radio operators. It is preparing “the community” to take responsibility for itself so that in an emergency we can look after our neighbours, we know what is available, we can get on doing it and not be a burden on the resources that will be required in a major emergency elsewhere. So we …train our people… in how to operate this facility in an emergency.

(Bill Rawiri, Ngā Hau e Whā Nga o Paparārangi, *Film for Change Aotearoa*, 2015 [71])

New Zealand’s capital city, Wellington is built on or near several major fault lines. The Wellington fault, which runs directly under Wellington City and transects the Hutt Valley to the north, is thought to be capable of generating an earthquake of magnitude 7.5 or greater on the Richter Scale. GNS Science (Institute of Geological and Nuclear Sciences Ltd.) has estimated that there is a 10% chance of a major rupture occurring on this fault within the next 100 years. There are at least four other active fault lines in the subduction zone under the greater Wellington region that are also capable of generating a significant seismic event. The Canterbury, and subsequent earthquakes closer to Wellington, have heightened awareness of earthquake vulnerability within the Wellington region as well as the need for disaster preparedness within the community [72]. As part of a wider disaster preparedness strategy, Ngā Hau e Whā o Paparārangi has developed programmes that facilitate community empowerment through strengthening community networks and social cohesion locally. Initiatives include weaving, cooking, gardening, rongoa (Māori medicine) and Māori language classes which are held at the marae. Additional linkages into the local school, retirement home and the parole office ensure that all of the community is involved in the delivery of the programmes strengthening the resilience of the community through familiarity with the marae and each other:

We have gone from a rickety old whare [house] to having this building completely refurbished. We have gone from having no Civil Defence emergency resilience programme, to having container loads of material out there, gone from having no money in the bank, to money in the bank, gone from having toxic land that we are restoring now and we have programmes operating with “the community” and the involvement of everybody around us, it is just stunning. The whole thing is community involvement, and is *by the people*, *for the people*, *owned by the people*, nga hau e wha [Ngā hau e whā refers to the people of the four winds, in the Māori world this a term that means including everybody] so it is all theirs and sort of 10 min from the Capital and Parliament.

(Bill Rawiri, Ngā Hau e Whā o Paparārangi, *Film for Change Aotearoa*, 2015 [71], *italics our emphasis*)

In this example of a community development approach the problem and subsequent actions to facilitate local disaster preparedness were identified by the marae community as a result of feeling ill-equipped to support traumatised evacuees from Christchurch following the February 2011 earthquake. The community itself, rather than individuals who happen to be present within a community setting, is the target of the intervention. Actions are focused upon capacity building through emergency response training and facilitating community resilience through outreach programmes that get diverse parts of the community working together. The local retirement home, primary school and disability community are invited to participate as evacuees in the welfare centre activation exercises which are held twice yearly at the marae. These linkages ensure that relationships with key people within the community, such as the school principal and retirement home manager, are maintained. The marae initiatives such as the community vegetable garden and cooking classes also enable the marae to identify local low income families that may be in need of greater support in the aftermath of a disaster. The programmes run at Ngā Hau e Whā o Paparārangi implicitly recognise that for indigenous people culture and cultural experience is crucial to the development and maintenance of good health [73]. Loss of culture is a threat to health as well as to resilience within Māori communities, culture therefore has the potential to become a positive resource for health gain as well as disaster risk mitigation [25,73]. 

Ngā Hau e Whā o Paparārangi is a member of Te Piringa o te Awakairangi which is a network of 10 marae located in the greater Wellington Region. Each of the marae within the network has developed similar emergency response training and outreach programmes within their communities. The network has developed linkages with Te Puni Kōkiri (the government department in charge of Māori affairs), the Wellington Regional Emergency Management Office, Red Cross and the Joint Centre for Disaster Research at Massey University. Te Piringa o te Awakairangi is now firmly established as a key component of the disaster preparedness and response network within the Wellington Region. This marae resilience network is an exemplar of a bottom-up community-development approach as it did not develop from the actions of local or regional emergency managers, it arose out of spontaneous community action and has community buy in that ensures its sustainability long term [74]. Subsequent to the formation of the network, Wellington regional emergency managers have recognised how the marae are able to add value in an emergency. They currently provide ongoing support to the initiative through attending meetings as a network partner, facilitating the provision of further resources (such as water storage tanks), providing advice and training to the community and evaluating their welfare centre activation exercises. Te Piringa o te Awakairangi is an example of how indigenous populations, that are often “marginalized” may be included in disaster preparedness, planning and response. 

### 3.6. Hard-to-Reach Populations or Hard-to-Reach Organisations?

The term “*hard-to-reach*” *populations* refers to groups within the population that are difficult to involve in public health programmes due to geographical location, social and economic circumstances, social isolation, cultural separateness and/or exclusion [10,75]. Within public health, migrants, indigenous peoples and floating populations that are socially invisible such as homeless, stigmatised, impoverished or illiterate groups are commonly identified as hard-to-reach [10,75]. An alternative view is to turn the lens away from a focus upon the group characteristics of hard-to-reach individuals to consider why the health system may be failing the most vulnerable people in society [37,76]. Research conducted in the UK that accessed mortality records and matched them against personal health information identified that over 80% of individuals who would typically be regarded as hard-to-reach had accessed medical care in the year prior to their death. This finding led the researchers to question whether for certain groups it was health services that were hard-to-reach rather than the populations themselves [77]. New Zealand literature has also identified that for Māori and Pacific peoples, health services are “hard-to-reach”. Māori present at the same rate as non-Māori for specialist health services, have higher mortality rates but receive fewer interventions for cardio-vascular disease [11] and colon cancer [78] compared to their European counterparts. 

A Christchurch initiative that developed following the Canterbury earthquakes as a result of hard to reach services for the elderly residing in the most deprived suburbs is also an example of those with the least resources collaborating to provide access to care. Ngāi Tahu elders identified a lack of accessible social support and acceptable healthcare services for elderly residents in Eastern Christchurch during the aftermath of the 2010–2011 Canterbury earthquakes. Hardship created by disruption to transport infrastructure post-impact was further compounded by the closure of health and social services and/or their migration to other areas of Christchurch due to severe seismic damage to facilities located in the hardest hit Eastern suburbs. Elderly Māori residents in Eastern Christchurch with health issues, and limited socio-economic resources were further marginalised through isolation in unsafe and unsecure homes. Ongoing isolation had an adverse impact on the residents’ psychosocial and functional wellbeing as well as physical health. In response, five Māori women community leaders collaborated to ensure elderly residents were able to access social, healthcare and material support. Although initially targeting the needs of elderly Māori, the initiative was rapidly expanded to address support issues faced by the wider elderly community in Eastern Christchurch. The communitarian approach to addressing the needs of the elderly also received international recognition, as the United Nations International Strategy for Disaster Reduction (UNISDR) documented the initiative as an exemplar of best practice [79]. Key factors in the initiative’s success have been the Māori women’s collectivised approach to leadership as well as the enactment of traditional cultural values and communitarian practices. These “grass roots” actions offer a counterpoint to more commonly applied and hierarchically structured community-based and expert-led approaches to addressing community needs in disaster contexts. Grass roots community-development initiatives tend to achieve longevity compared to top-down community-based programmes. Five years later, and at the time of writing, this initiative is still on-going in Christchurch. 

Within the disaster literature vulnerable populations include migrant [80] and indigenous people, [81] the elderly, [82] children, medically dependent persons, homeless or shelter dependent people, physically or mentally disabled individuals and those who are rurally isolated [83]. In the past, emergency response planning developed in isolation from the community resulting in emergency managers having to make decisions that impacted upon groups with which they were unfamiliar [67]. Insular approaches to emergency management also meant that potentially useful collaborations with community groups were overlooked. Reflecting upon the historical command and control nature of emergency management prevalent in the 1990s one Ngāi Tahu Kaumātua (elder) described Civil Defence using language that identifies the organisation as “hard-to-reach”. This elder was a participant in the Māori research and had previously held a senior role liaising with the government in regards to Māori welfare issues said:

way back in … 1993 there was [Cyclone] Bola, the Tairawhiti [and] Edgecumbe earthquakes and I said to Civil Defence in Wellington… the places for the disaster areas, the sector posts ought to be every marae in this country! …You people, you want to put people into the school or church, where … [are] the mattresses, …the cooking facilities,… the toilet facilities? So it’s time you people recognise that the marae is the only place in this country for sector posts… I’ve been saying that for years … [But] No because they want to keep it to themselves….

(CA, 2012)

Ngāi Tahu comprise the largest group within the Canterbury Māori community [84], and as the resident tribe, have a cultural imperative to ensure the wellbeing of all residents in the region. After the 22 February, earthquake in 2011, the Iwi (tribe) initiated a meeting with Māori representatives from government, private organisations and other tribes in order to collaboratively develop a coordinated earthquake response [25]. With the noted exception of representatives from the civil defence and emergency management infrastructure, the meeting was well attended by representatives from the Urban Māori Authority, the Ministry of Māori Development, Māori parliamentarians, the New Zealand Police, Non-Government Organisations (NGOs) and other tribes [85]. A national Māori Recovery Network was established that exhibited characteristics that are consistent with a community development approach to disaster risk reduction, including collaborative accountability, authority, agency, and actions. Ngāi Tahu as regional guardians undertook the response leadership role, providing logistical governance and coordination of community support. The Iwi subsequently communicated and negotiated decision-making with NGOs, other iwi (tribes) Māori stakeholder institutions, as well as central government and local authorities [86]. The nationalised Māori Earthquake Recovery Network was highly effective and provided support in the form of shelter, food, water, clothing medical and social services to approximately 20,000 households. However, coordination with the formal emergency management infrastructure was initially extremely problematic [61]. It took eight days for the Māori response leadership to establish direct communication and a relationship with the emergency management infrastructure. This access had to be negotiated through a corporate side party, engaged in lifelines logistics [31].

…It was a bit slow in us [the Māori Recovery Network] getting involved with the authorities, in fact it took us 8 days to break in… [And]… from that single meeting we then had a link directly to Civil Defence, so every day from then on all our reports went to Civil Defence….

(SMS)

Following the 22 February earthquake central government took over co-ordination of the local emergency response in Christchurch. The imposition of external control disconnected a wide range of local relationships including ties to local Māori. The Māori Recovery Network was linked into Eastern communities that were severely impacted by the earthquakes. Forms of flax roots (grass roots) reporting meant that the network had important information about local conditions as well as unmet needs. The delayed coordination of the Māori response with the formal disaster and emergency management infrastructure contributed to duplication or the absence of services, such as fresh water delivery and portable toilets, in some regions [55]. The Chairperson of Te Rūnanga o Ngāi Tahu subsequently communicated and negotiated decision-making with NGOs, Northern iwi (tribes), as well as Government and Local authorities in order to ensure that the Māori Recovery Network coordinated effectively with the formal disaster management infrastructure [87]. Within the public health and disaster literature indigenous populations are constructed as vulnerable and/or “hard-to-reach” [39,88,89,90,91]. The Ngāi Tahu response to the earthquakes disrupts that narrative, and provides an example of how the development of local linkages and networks prior to a disaster ensures that lines of communication are open and that organisations involved in the formal emergency response are not “hard-to-reach”. 

### 3.7. The Prevention Paradox

*The prevention paradox* [24] states that a focus on reducing risk across the population will have little benefit for the individual, while a sole focus on targeting at risk individuals or groups will have a limited effect at the population level. The prevention paradox may be used to understand and reduce risk as well as to facilitate readiness within the “new” Disaster Risk Management field. 

Public health action focuses on the whole population, rather than at-risk individuals and groups [34]. The risk of disease is distributed across a population with the pattern of distribution normally resembling a bell curve. That is, there is a small proportion of people at high-risk, however the majority of people in a population are at low or medium risk. Compared to the relatively small number of people who are at high risk, more cases of disease will occur among the large proportion of the population at low or medium risk. Traditional approaches that focus upon those at most risk are based on the assumption that this is a distinct group requiring specially targeted interventions. A targeted approach is seductive as it appears to be highly appropriate and timely for individuals and seems to offer effective use of limited resources. However, Rose argues that focusing on high-risk individuals considers only the margins of the problem, and potentially misses a large proportion of the population who may still have poor outcomes [24]. This is known as the “prevention paradox”. According to Rose strategies aimed at reducing risk across the whole population will be more effective in improving population health [24]. A whole population approach also avoids negative and harmful effects through labelling at-risk groups. A population based approach considers the social determinants of health, taking into account how individuals’ attitudes and behaviours are shaped by social and environmental factors. Directing action at more upstream risk factors aims to change the underlying causes of disease and includes actions such as legislation, or healthy transport policies. This approach has the greatest potential to increase overall population health [24]. A structural or population based approach underpins priority three within the Sendai Framework [2] which focuses upon investing in disaster risk for resilience. This action area proposes using legislative change, fiscal measures, strengthening of critical infrastructure, revision of building codes as well as mainstreaming disaster risk management within land use planning. 

According to the prevention paradox disaster risk can be reduced through actions that focus on the whole population prior to a disaster, rather than solely on at-risk individuals and groups post-impact. The prevention paradox may be illustrated through returning to the disability accessible social housing example discussed previously in the section on inequality and inequity. Disaster risk is normally distributed across a population with a small number of people being recognised as high risk. The disabled and elderly are a high risk group that is vulnerable to displacement and subsequent acute housing need following a disaster [40,92]. Ensuring that disability accessible social housing is earthquake resilient and that the houses are well maintained would reduce risk by increasing housing security post-impact amongst the targeted disabled population. New Zealand’s housing stock is of poor quality, compared to that of other OECD countries [93]. Prior to the 2001–2011 earthquakes, the majority of the rental housing stock in Christchurch was old, poorly maintained and constructed when building standards and techniques did not factor in a need for earthquake resilience. In addition, a higher prevalence of poor housing is located within the privately rented sector compared to other tenures. The state of the rental housing stock in Christchurch meant that the absolute number of people who were displaced following the 22 February 2011 earthquake was greater among the general population of renters. The prevention paradox states that focusing on high-risk individuals considers only the margins of the problem, and potentially misses a large proportion of the population who may still have poor outcomes post-disaster. 

An upstream focus on using legislation to improve all rental housing would reduce the absolute risk of displacement across the entire population including people with disabilities. Legislative action, such as introducing a rental warrant of fitness [93], would improve the standard of rental housing across a large and increasing proportion of the population who are renting resulting in better health outcomes. More robust housing would, in turn, strengthen community resilience and reduce demand on emergency services and welfare centres in the aftermath of a major disaster. Legislative action to strengthen the building code so that dwellings are earthquake resilient will only bring outcome benefits to individuals in the event of a major disaster. On the other hand, providing earthquake resilient housing to only high risk individuals and groups will have a limited effect on ensuring housing security and health benefits at the population level in a major disaster. Priority area four within the Sendai Framework [2] which focuses upon “Build Back Better” post-disaster aims to direct action during the reconstruction phase at more upstream risk factors in order to change the underlying causes of disaster vulnerability within the built environment. Actions within this approach include legislative change, land use and urban planning policies [94] as well as strengthening lifelines and infrastructure [72]. This method has the greatest potential to reduce the distribution of disaster risk across the entire population and thereby improve outcomes following future disasters. 

### 3.8. The Inverse Prevention Law

The prevention paradox has been recently extended to include the inverse prevention law. The *Inverse Prevention Law* [12] states that “those in most need of benefitting from preventive interventions are least likely to receive them” (p. 190) [95]. Health promotion programmes that successfully improve overall population health outcomes may increase disparities in health between groups. Smoking rates, for example have decreased over time in many Western countries. However, the quit rate has been greater among people in higher socio-economic groups creating disparities in health damaging behaviours and outcomes between advantaged and disadvantaged groups within the same population [96]. *Intervention generated inequalities* occur when there are disparities in service provision, response, access, uptake, compliance and long term sustainability between socio-economic groups [95,96]. Downstream interventions that require voluntary action by individuals such as health education or mass media campaigns are examples of interventions that increase inequalities. Upstream or structural interventions at the policy level, that focus upon delivering benefits to disadvantaged groups as well as reducing inequity, are less likely to increase inequalities and may even reduce them. Examples of public health interventions that do not increase inequalities include reducing price barriers, improvements to workplace health and safety legislation or fiscal measures such as tobacco pricing [97].

The promotion of “national strategies to strengthen public education and awareness for disaster risk reduction” (p. 10) through social media is a focus of priority one within the Sendai Framework which emphasizes understanding disaster risk [2]. In New Zealand mass media campaigns such as “Drop, Cover and Hold” [98] and “Fix, Fasten, Forget” [99] are the preferred means for raising awareness about earthquake preparedness and response among the general population. Mass media campaigns are useful for changing attitudes towards emergency preparedness so that people working within the area of disaster risk reduction are able to argue for greater resources as well as positive legislative change. However, evidence from public health has identified that mass media campaigns are rarely effective among socio-economically deprived populations [96]. Drawing upon the inverse prevention law principle we suggest that people in higher socio-economic groups are more likely to respond to and comply with disaster preparedness messages creating disparities in outcomes post disaster between advantaged and disadvantaged groups within the same population. Intervention generated inequalities may also appear between the general population and people with a disability when preparedness information is aimed at the able-bodied population. 

Disabled people who were living in Christchurch at the time of the Canterbury earthquakes identified that advice provided by Civil Defence Emergency Management was not appropriate to their situation, as it was too general or made assumptions about people’s bodies or lives that did not apply to them [30,53]. Shane, a participant in the disability research who has profound hearing loss and is an advocate for the deaf community, made the following comment about emergency preparedness information following the magnitude 7.1 September 2010 earthquake:

…Round about November [2010] we started preparing ourselves [for a future seismic event]… I found Civil Defence completely useless… because it’s not designed for people with a disability.

(Shane, 2012)

Disaster risk is also known to be socially patterned and disproportionately prevalent in disadvantaged communities [41,100,101]. Intervention generated inequalities are likely to be magnified by preparedness strategies that will achieve a disproportionate uptake by able-bodied individuals and/or those in higher socio-economic groups through excluding the disabled population, requiring co-funding and/or voluntary action by individuals. The Sendai Framework [2] recommends that national campaigns involving public education to raise awareness of disaster risk should take into account “specific audiences and their needs” (p. 10). Without targeting those areas and groups where socio-economic disparities are greatest, disaster risk reduction strategies may fail to reach the areas and people that need it most. In unequal societies, statutory regulation, combined with population based strategies that reduce inequity, may be a more effective way to strengthen community resilience than disaster risk reduction strategies in which individual behavior is the target of intervention. 

## 4. Conclusions

This paper has outlined relatively recent changes within the disaster risk management field, towards the inclusion of risk reduction and building resilience. This resonates with public health’s focus on social determinants of health, moving to a broader focus which acknowledges these key conditions can shape population outcomes following disaster. Research highlights that following disasters, low socioeconomic households and communities face increased risk and amplified lack of access to the social determinants of health. Similarly, there is evidence of inequalities in outcomes following disasters, some of which can be defined as inequities as they are either avoidable or unfair. Inequitable outcomes present in the aftermath of a disaster, particularly for already deprived population groups, often stem from existing structural inequalities. Māori initiatives following the Canterbury earthquakes exemplify how population outcomes can be improved through equity-driven approaches, which focus on building community resilience through reducing vulnerability. The inequitable patterning of outcomes post-disaster can also be linked to the distribution of resources post-impact and in recovery. The experiences of deprived neighbourhoods in Canterbury in receiving less attention and resources than wealthier suburbs illustrate the inverse care law for those positioned outside mainstream responses. 

The Sendai Framework directs more action upstream, to influence the determinants of disaster vulnerability as disaster risk is socially patterned and disproportionately experienced in disadvantaged communities. Public health maintains concern with the population as a whole, and combines diverse strategies necessary when acknowledging the interconnected relationships of determinants across different levels (micro, meso, and macro levels of social systems). Traditional approaches focusing at the individual level on risk and literacy within public health and the disaster fields shows that education and awareness does not guarantee that individuals respond appropriately or adequately. Presumptions that once “fully” informed, individuals and communities will change their behaviour exaggerates the ease of behaviour modification and risks blaming and stigmatising individuals. The “new” disaster risk management field, aligns more closely with the “new” public health, in recognising that micro-level preparedness activities must be used in conjunction with other approaches. Directing action at the community level can help to ensure disaster initiatives respond to the needs of all, within the context of their lives, while engaging communities in disaster risk reduction and response may also increase the resources that facilitate disaster resilience. This application of socioecological approaches facilitates inter-sectoral action, and builds on the physical and economic resources of the communities to increase their resilience and autonomy. Community development approaches incorporate action on social, economic and environmental factors which influence individuals’ behaviour, and can be more effective in addressing health inequities. This move away from individual and lifestyle approaches to community capacity within the disaster field has focused on the everyday contexts in which people live and is, perhaps, particularly important in unequal societies. Community action may be a more effective way to strengthen community resilience than disaster risk reduction strategies in which individual behavior is the target of intervention. Structural approaches, which identify macro level determinants resulting in poor outcomes and health inequity, are also more comprehensive than individual lifestyle approaches. Improving outcomes following disasters requires changing environments, policies and regulations to support equity and resilience. 
